# Sitagliptin Accelerates Endothelial Regeneration after Vascular Injury Independent from GLP1 Receptor Signaling

**DOI:** 10.1155/2018/5284963

**Published:** 2018-02-08

**Authors:** Friederike Remm, Nicolle Kränkel, Daniela Lener, Daniel J. Drucker, Sieghart Sopper, Christoph Brenner

**Affiliations:** ^1^Department of Internal Medicine III, Cardiology and Angiology, Medical University of Innsbruck, Innsbruck, Austria; ^2^Department of Cardiology, Charité-Universitätsmedizin Berlin, Campus Benjamin Franklin, Berlin, Germany; ^3^DZHK (German Center for Cardiovascular Research), Partner Site Berlin, Berlin, Germany; ^4^Lunenfeld-Tanenbaum Research Institute, Mt. Sinai Hospital, University of Toronto, Toronto, ON, Canada; ^5^Department of Internal Medicine V, Hematology & Oncology, Medical University of Innsbruck, Innsbruck, Austria; ^6^Department of Cardiology, Reha Zentrum Muenster, Münster, Tirol, Austria

## Abstract

**Introduction:**

DPP4 inhibitors (gliptins) are commonly used antidiabetic drugs for the treatment of type 2 diabetes. Gliptins also act in a glucose-independent manner and show vasoregenerative effects. We have shown that gliptins can remarkably accelerate vascular healing after vascular injury. However, the underlying mechanisms remain unclear. Here, we examined potential signaling pathways linking gliptins to enhanced endothelial regeneration.

**Methods and Results:**

We used wild-type and GLP1 receptor knockout (*Glp1r*^−/−^) mice to investigate the underlying mechanisms of gliptin-induced reendothelialization. The prototype DPP4 inhibitor sitagliptin accelerated endothelial healing in both animal models. Improved endothelial growth was associated with gliptin-mediated progenitor cell recruitment into the diseased vascular wall via the SDF1-CXCR4 axis independent of GLP1R-dependent signaling pathways. Furthermore, SDF1 showed direct proproliferative effects on endothelial cells. Excessive neointimal formation was not observed in gliptin- or placebo-treated *Glp1r*^−/−^ mice.

**Conclusion:**

We identified the SDF1-CXCR4 axis as a crucial signaling pathway for endothelial regeneration after acute vascular injury. Furthermore, SDF1 can directly increase endothelial cell proliferation. Gliptin-mediated potentiation of endothelial regeneration was preserved in *Glp1r*^−/−^ animals. Thus, gliptin-mediated endothelial regeneration proceeds through SDF-1/CXCR4 in a GLP1R-independent manner after acute vascular injury.

## 1. Introduction

Gliptins are inhibitors of the dipeptidyl peptidase-4 (DPP4) enzyme and have demonstrated a vasoprotective and vasoregenerative impact after endothelial injury and during early atherosclerosis. Various preclinical and clinical studies have confirmed these favorable effects [1–6]. Several gliptins are clinically approved as antidiabetic drugs for the treatment of type 2 diabetes mellitus (T2DM). However, the underlying mechanisms of their vasoregenerative effects are not completely understood [7]. Some clinical trials have confirmed a good safety profile for the use of gliptins in diabetic [8–10] and nondiabetic patients [11]. Together with our previously published preclinical data, gliptins appear to be promising agents for the treatment of vascular diseases.

Several pathways and substrates may explain the positive gliptin-mediated effects on the vascular system. These include the inhibition of glucagon-like peptide 1 (GLP1) and stromal cell-derived factor 1 (SDF1) degradation. The latter is responsible for the recruitment of circulating progenitor cells (ciPC) via the SDF1-CXCR4 pathway [3, 12–15].

In our previous studies, we have shown that sitagliptin can enhance endothelial regeneration after vascular injury. We verified the SDF1-CXCR4 signaling as the underlying regenerative cellular mechanism. SDF1, a physiological DPP4 substrate, is expressed by adhesive thrombocytes, vascular smooth muscle cells, and activated endothelial cells [16–18]. SDF1 binds to CXCR4 on the surface of ciPC and recruits them to the sites of vascular injury. Here, ciPC can induce resident endothelial cell proliferation via paracrine mechanisms in the border zone of the injured area. Gliptins prevent cleavage of SDF1 by inhibiting DPP4 activity, which leads to an increase of local SDF1 concentrations [3, 19, 20]. Nevertheless, inhibition of DPP4 degradation with sitagliptin does not lead to progenitor cell recruitment in atherosclerotic lesions. The protective impact of gliptin treatment led to the priming of monocyte differentiation towards cholesterol-exporting M2 macrophages. These cells can inhibit progression of atherosclerotic plaque formation, which explains the sitagliptin-mediated vasoprotection in chronic endothelial injury [2].

The incretin GLP1 is another important DPP4 substrate, which is inactivated by N-terminal enzymatic cleavage [21]. Besides its function in glucose metabolism, GLP1 might have additional protective and regenerative effects on the vascular system. These could be explained by its positive influence on endothelial function, blood lipid profiles, and vascular inflammation [13, 22–24]. Since both SDF1 and GLP1 are DPP4 substrates, pharmacological inhibition of DPP4 leads to an increase of circulating levels of both active GLP1 and SDF1. To dissect the contributions of gliptin-based signaling pathways, we used *Glp1r*^−/−^ mice and investigated the relevance of GLP1 receptor signaling in endothelial regeneration.

## 2. Material and Methods

### 2.1. Scratch Assay

For the evaluation of endothelial cell proliferation, human umbilical endothelial vein cells (HUVECs; Promo Cell, Heidelberg, Germany) were grown until they formed a cellular monolayer. After creating scratches with a 100 *μ*l pipette tip, the cells were incubated with recombinant human CXCL12/SDF1 (R&D Systems, Minneapolis, USA) in different concentrations (0 ng/ml, 1 ng/ml, 10 ng/ml, and 100 ng/ml) SDF1 for 24 h. Subsequently, photos were taken with a light microscope (Leica, Wetzlar, Germany).

### 2.2. DPP4 Activity Assay

To perform DPP4 activity measurements, we used a DPP4 Activity Fluorometric Assay Kit following the manufacturer's instructions (BioVision, Milpitas, USA). Serum was taken from *Glp1r*^−/−^ mice after three days of oral sitagliptin administration (*n* = 4 (pooled), 3 mice each sample). Serum from placebo-treated mice collected at the same point served as reference. DPP4 activity measurements using the Fluorometric Assay Kit were previously established by our group and verified using mass spectrometry [3].

### 2.3. Animal Experiments

All animal experiments were approved by the Austrian Federal Ministry of Science, Research and Economy (authorization number BMWFW-66.011/0160-WF/V/3b/2014). We used *Glp1r*^−/−^ mice with a C57Bl/6N genetic background in our experiments. *Glp1r*^−/−^ mice emerged from an in-house breeding originating from 9 female and 4 male *Glp1r^−/−^* mice (Taconic, Lille Skensved, Denmark). Dr. Daniel Drucker (Lunenfeld-Tanenbaum Research Institute, Toronto, Canada) created the *Glp1r*^−/−^ animals and provided them for our experiments [25, 26]. C57Bl/6 mice were delivered by a commercial breeder (Charles River, Sulzfeld, Germany).

### 2.4. Administration of Sitagliptin and AMD3100

To ensure a dose of 500 mg/kg/d sitagliptin, we fed a premixed chow containing 2550 mg sitagliptin per kg chow (Ssniff Spezialdiäten, Soest) to the animals ad libitum. We calculated the sitagliptin food concentration based on an average daily food intake of 4.9 g/day/mouse as previously described [2, 3, 27].

For the administration of the CXCR4 blocker AMD3100 (1.25 mg/kg/d; Sigma-Aldrich, St. Louis USA), we chose subcutaneous injections. We have previously determined an optimized dose that is able to block the CXCR4 receptor on circulating progenitor cells without mobilizing progenitor cells from bone marrow [27].

### 2.5. Carotid Injury and Quantification of Endothelial Regeneration

We induced endothelial lesions in the common carotid arteries as previously described [28] under isoflurane anesthesia. In brief, we induced a 4 mm long endothelial injury of the left common carotid artery in 9–12-week-old mice (*n* = 9/group) using a bipolar microregulator (Vio50C, ERBE Elektromedizin, Tübingen, Germany) with a power of 2 W for 0.5 seconds at the left common carotid artery. Randomization allocated the mice into four treatment groups: placebo (Plac), sitagliptin (Sita), AMD3100 (AMD), and sitagliptin and AMD3100 (SitaAMD).

Three days after carotid injury, we evaluated reendothelialization by the quantification of the regenerated area using Evans blue staining as previously described [3]. Shortly, we injected 100 *μ*l of a 5% Evans blue solution (Sigma-Aldrich, St. Louis, USA) to the anesthetized mice via the tail vein and scarified the mice 2 minutes later for harvesting the common carotid artery. Evans blue stained the remaining deendothelialized area. We calculated the reendothelialization as the reendothelialized area divided by the area of initial injury [3].

### 2.6. Flow Cytometry Analyses

Three days after carotid injury, carotid arteries were subjected to flow cytometry-based characterization using a protocol modified from an earlier described one [3], in order to increase detectability of the ciPC. Briefly, we harvested the left (selective injured area) and right (uninjured) carotid arteries and minced the vessels with microsurgical scissors. To obtain a sufficient amount of cells, we pooled two arteries per sample (*n* = 8/group) and incubated them in a 0.2% collagenase (Worthington) and 0.01% DNAse (Roche, Basel, Switzerland) solution 45 minutes for digestion. After washing in PBS, we stained the single-cell solution with the following antibodies for the detection of ciPC: PE-anti-CXCR-4 (BD Pharmigen, Heidelberg, Germany), BV421-anti-CD133 (BioLegend, San Diego, USA), APC-anti-Flk-1 (BD Pharmigen), and V500-anti-CD45, and analyzed with an LSRFortezza flow cytometer (BD Biosciences) [3, 29, 30]. The previously established antibody staining was refined by an extensive antibody titration protocol [3]. Furthermore, we used antibodies against F4/80, Gr1, and CD206 (PE-F4/80, BV421-Gr-1, and APC-CD206; all BioLegend and V500-CD45; BD Horizon) for the detection of M1 and M2 macrophages [2].

### 2.7. Histological Evaluation of Neointimal Formation

For the histological evaluation of gliptin-mediated effects on neointima formation after endothelial injury, we sacrificed mice (*n* = 8/group) 28 days after carotid injury. Sitagliptin- and sitagliptin and AMD3100 treatments were provided in this experiment over a period of 6 days after acute endothelial injury. We then cut the Tissue-Tek® O.C.T. compound-embedded (Sakura Fintek, Torrance, USA) carotid arteries into 5 *μ*m sections (6 to 8 per mouse, cryo-sections). For the Weigert's Elastica van Gieson staining of the serial sections, we used a commercial staining kit (DiaPath, Martinengo, Italien) and analyzed neointimal formation by measuring the sections planimetrically. Finally, we calculated the “intima (I)-to-media (M) ratio” and the “intima + media to total cross section (tcs),” “I/I + M,” and “(I + M)/tcs”.

### 2.8. Statistical Analyses

For the estimation of the sample size for the experiments (reendothelialization, flow cytometry, and histology), we used “Power and Sample Size Program version 3.1.2, July 2014” [31]. We used ImageJ software for the morphometric analyses, and GraphPad Prism software (version 7) for statistical analyses. The results are expressed as means with standard error, and normality and variances of equality are confirmed for multiple group comparisons. One-way-analysis of variances (ANOVA) followed by the Bonferroni correction was used for multiple group comparisons, and for the comparison of two groups, we used unpaired Student's *t*-test. Differences were regarded significant at a *p* value of ≤0.05.

## 3. Results

### 3.1. Sitagliptin Inhibits DPP4 Activity in *Glp1r*^−/−^ Mice

**Figure 1:**
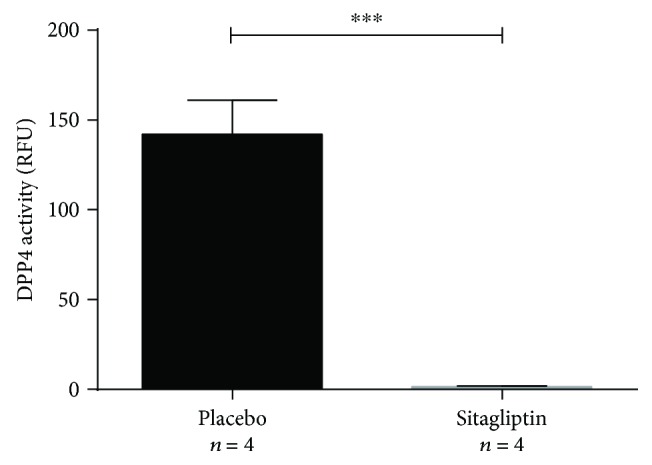
Administration of oral sitagliptin over a period of three days significantly reduced serum DPP4-activity (^∗∗∗^*p* ≤ 0.001).

We first confirmed that sitagliptin administration of 500 mg/kg/d led to a sufficient inhibition of DPP4 activity in *Glp1r*^−/−^ mice compared to untreated controls. Sitagliptin administration led to a reduction of DPP4 activity from 141.92 ± 19.11 relative fluorometric units (RFUs) to 1.22 ± 0.71 RFUs (Figure 1).

### 3.2. Quantification of Endothelial Regeneration

**Figure 2:**
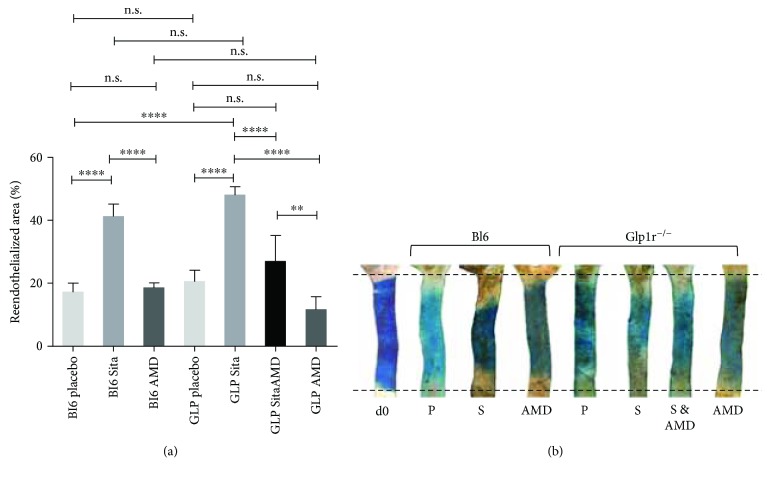
(a) Sitagliptin treatment significantly improved endothelial regeneration compared to placebo treatment in both, Glp1r^−/−^ (GLP) and C57Bl/6N wild-type (Bl6) mice. Additional treatment with the CXCR4 receptor blocker AMD3100 abolished the positive gliptin effect completely (n.s. = not significant; ^∗∗^*p* ≤ 0.01 and ^∗∗∗∗^*p* ≤ 0.0001). (b) Evans blue staining of the injured carotid arteries three days after carotid injury (d0 = day 0; P = placebo; S = sitagliptin; AMD = AMD3100; and S & AMD = sitagliptin and AMD3100).

Three days after carotid injury, we observed accelerated endothelial regeneration in the sitagliptin-treated mice (*Glp1r*^−/−^ and C57Bl/6N). In the *Glp1r*^−/−^ animals, sitagliptin led to a significantly larger reendothelialized area (r.a., 48.0% ± 2.61) than placebo treatment (20.5% ± 3.57 r.a.). Sitagliptin-treated wild-type mice also showed significantly better vascular regeneration (41.1% ± 4.05 r.a.) as compared to wild-type placebo group (17.2% ± 2.86 r.a.; Figures 2(a) and 2(b)) whereas *Glp1r*^−/−^ mice did not show a quantitative difference in endothelial healing compared to the C57Bl/6 wild-type controls.

Cotreatment with the CXCR4 blocker AMD3100 completely abolished the sitagliptin-elicited improvement of endothelial regeneration in *Glp1r^−/−^* mice (26.9% ± 2.77 r.a.). However, AMD3100 alone did not significantly affect endothelial regeneration in the placebo-treated *Glp1r^−/−^* (11.55% ± 1.39 r.a.) and wild-type (18.52 ± 1.58) mice (Figures 2(a) and 2(b)).

### 3.3. Quantification of Circulating Progenitor Cells

**Figure 3:**
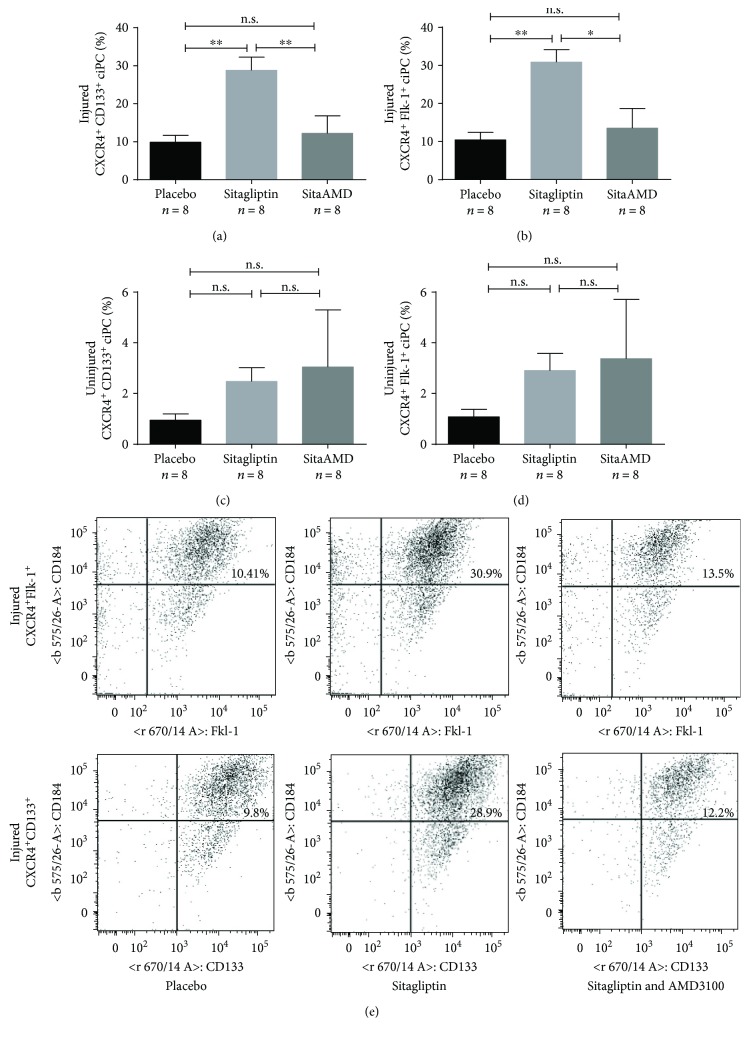
(a, b) The DPP4 inhibitor sitagliptin significantly enhanced the recruitment of CXCR4^+^CD133^+^ and CXCR4^+^Flk-1^+^ ciPC to the injured arterial walls. Additional administration of AMD3100 abolished the sitagliptin-mediated recruitment of ciPC (n.s. = not significant; ^∗^*p* ≤ 0.05; and ^∗∗^≤ 0.01). (c, d) The different treatments had no effect on the proportion of ciPC in the uninjured arterial walls (n.s. = not significant). (e) Representative dot plots from the FACS analyses.

Using flow cytometry analyses of the injured carotid arteries, we detected an increased recruitment of ciPC in sitagliptin-treated *Glp1r*^−/−^ mice compared to the placebo group. This was true for CXCR4^+^CD133^+^- (28.9% ± 3.41) and CXCR4^+^Flk-1^+^- (30.9% ± 3.27) recruited progenitor cells in the arterial wall of sitagliptin-treated mice compared to the placebo group (CXCR4^+^CD133^+^ 9.8% ± 1.84 and CXCR4^+^Flk-1^+^ 10.41% ± 1.96). Consistent with the reendothelialization assays, an addition of the CXCR4 blocker AMD3100 abolished progenitor cell recruitment in sitagliptin-treated *Glp1r*^−/−^ mice and resulted in the recruitment of 12.2% ± 4.64 CXCR4^+^CD133^+^ or 13.5% ± 5.22 CXCR4^+^Flk-1^+^ ciPC (Figures 3(a) and 3(b)). Uninjured carotid arteries showed a comparably low proportion of recruited progenitor cells that were independent from the treatment with sitagliptin or AMD3100 (see Figures 3(c) and 3(d)).

### 3.4. Neointimal Formation in Carotid Arteries

**Figure 4:**
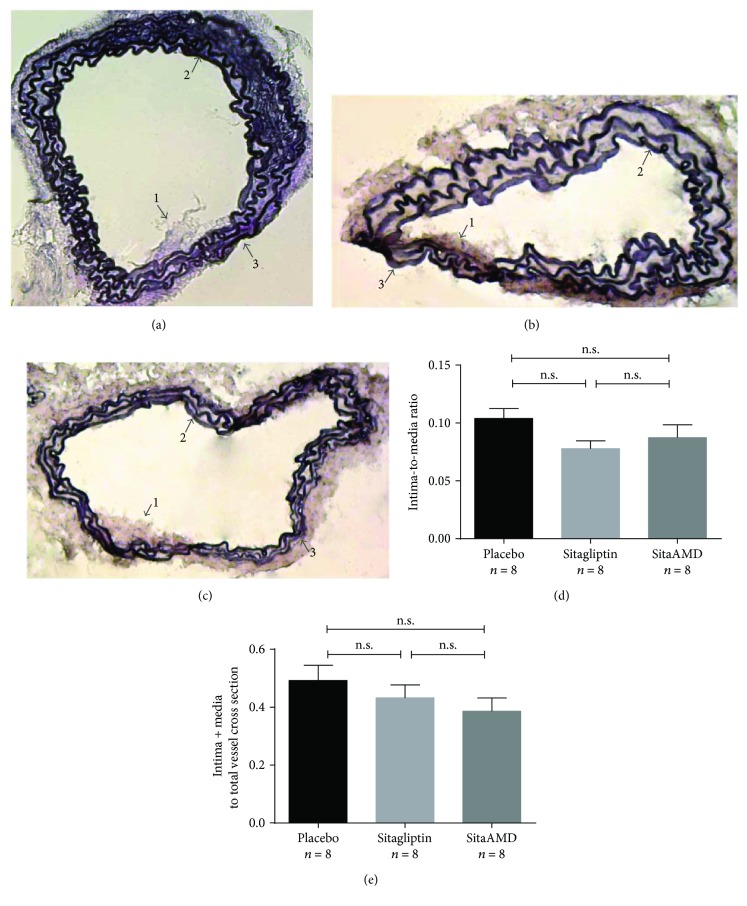
(a–c) Weigert's Elastica van Gieson staining of the injured carotid arteries 28 days after vascular injury. (d, e) “Intima-to-media ratio” and “intima + media to total vessel cross section” were not affected by the different treatments (n.s. = not significant).

28 days after endothelial injury placebo-, sitagliptin-, and sitagliptin and AMD3100-treated *Glp1r*^−/−^ mice showed no differences in the extent of neointima formation (Figure 4). Neither “intima-to-media ratio” (Plac 0.1 ± 0.008; Sita 0.08 ± 0.006; and SitaAMD 0.09 ± 0.011) nor “intima + media to total cross section” (Plac 0.49 ± 0.05; Sita 0.43 ± 0.04; and SitaAMD 0.38 ± 0.04) showed significant differences between the treatment groups.

### 3.5. Quantification of M1 and M2 Macrophages

**Figure 5:**
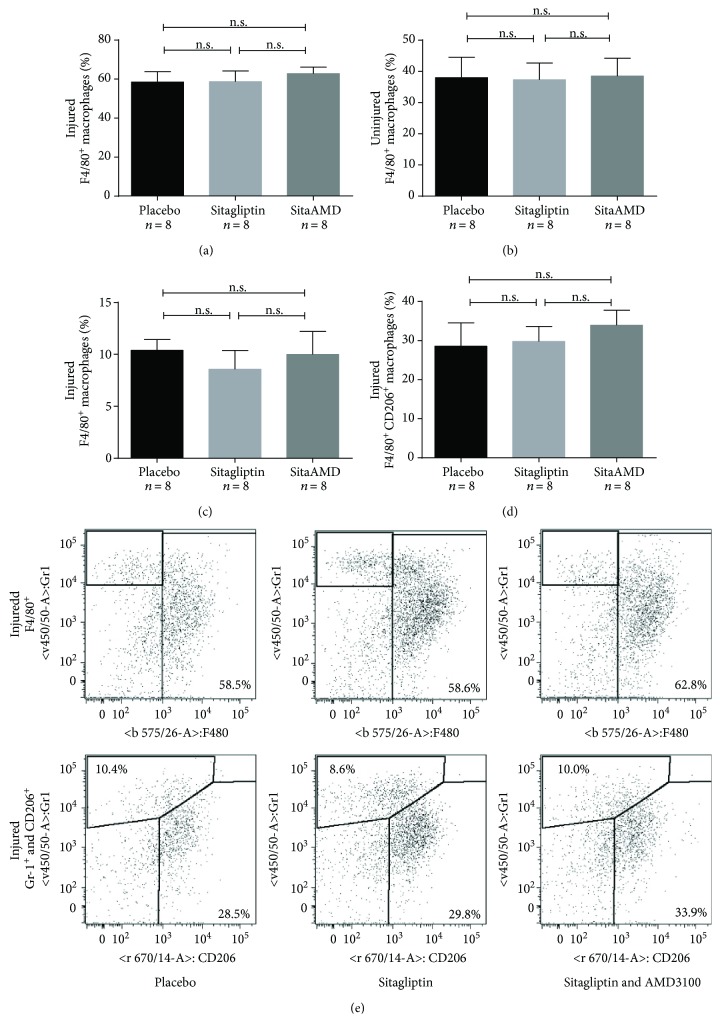
(a, b) Sitagliptin and sitagliptin + AMD3100 treatment had no significant influence on the total macrophage content in the injured and also in the uninjured carotid artery (n.s. = not significant). (c, d) The different treatments had no significant influence on the proportion of F4/80^+^Gr-1^+^ M1 and F4/80^+^CD206^+^ M2 macrophages (n.s. = not significant). (e) Representative dot plots from FACS analyses. Upper row shows F4/80^+^ total macrophages (right quadrant), and bottom row shows F4/80^+^Gr-1^+^ M1 (upper left quadrant) and F4/80^+^CD206^+^ M2 (bottom right quadrant) macrophages.

Sitagliptin treatment had no effect on total macrophage content or macrophage subtypes in the injured carotid artery wall of *Glp1r^−/−^* mice three days after carotid injury. The placebo group showed a total macrophage content (F4/80^+^ cells) of 58.5% ± 5.27 (injured vessel); the sitagliptin group showed 58.6% ± 5.55 F4/80^+^ cells, and the sitagliptin and AMD3100 group 62.8% ± 3.27. Analyses of the uninjured carotid arteries resulted in a minor proportion of F4/80^+^ macrophage content (Plac 37.9% ± 6.62; Sita 37.3% ± 5.38; and SitaAMD 38.5% ± 5.74) which also did not differ significantly between the groups (Figures 5(a) and 5(b)).

Furthermore, the proportion of inflammatory F4/80^+^Gr-1^+^ M1 (Plac 10.4% ± 1.04; Sita 8.6% ± 1.8; and SitaAMD 10% ± 2.23) and regenerative F4/80^+^CD206^+^ M2 macrophages (Plac 28.5% ± 6.04; Sita 29.8% ± 3.86; and SitaAMD 33.9% ± 3.84) in the injured carotid artery did not differ significantly between groups (Figures 5(c) and 5(d)).

### 3.6. SDF1 Exerts Direct Proproliferative Effects on Endothelial Cells

**Figure 6:**
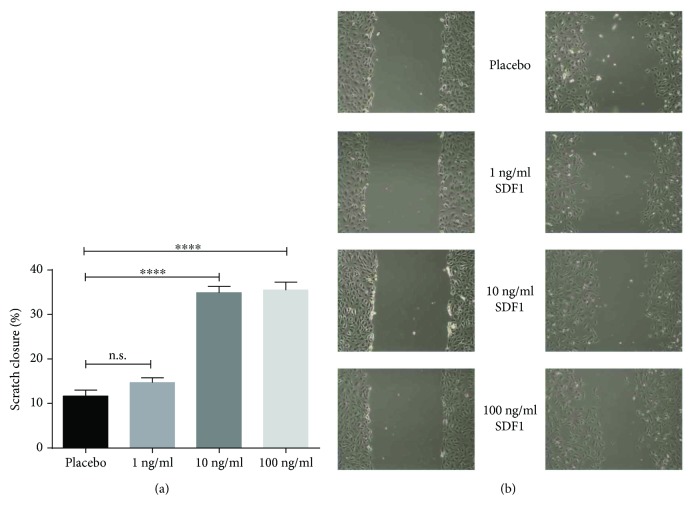
(a) 24 h incubation with 10 ng/ml and 100 ng/ml SDF1 leads to an increased scratch closure compared to 1 ng/ml SDF and placebo treatment (n.s. = not significant; ^∗∗∗∗^*p* ≤ 0.0001). (b) Representative images of the scratch size immediately after scratch induction (left) and after 24 h (right).

24 h after scratching, the HUVEC monolayer showed a significantly better scratch closure if incubated with SDF1 (10 ng/ml and 100 ng/ml). Compared to placebo control (11.6% ± 1.38 scratch closure), cells incubated with 10 ng/ml SDF1 showed a scratch closure of 34.9% ± 1.38 after 24 h. 100 ng/ml SDF1 led to a scratch closure of 35.5% ± 1.76. In contrast, the lower concentration of 1 ng/ml SDF1 only evinced a scratch closure of 14.7% ± 1.12 (Figure 6).

## 4. Discussion

DPP4 inhibitors mediate their vasoprotective impact via various molecular pathways and cellular mechanisms. Gliptins inhibit cleavage of the incretin GLP1 and the cytokine SDF1. While SDF1 acts via the SDF1-CXCR4 signaling pathway, GLP1 might mediate vascular protection via a GLP1R/cAMP-dependent activation of the AMPK/PI3K-Akt/eNOS pathway [32]. Both molecules are important potential factors in the diseased vascular system [33]. In previous studies, we have shown that DPP4 inhibition leads to an increase in local SDF1 concentration after endothelial injury. SDF1 improves the recruitment of regenerative progenitor cells which can mediate an accelerated vascular healing [3]. However, it is yet unclear whether concomitant inhibition of GLP1 cleavage may have supported this regenerative effect. Therefore, we further investigated vascular healing in a homozygous knockout mouse model lacking the GLP1 receptor.

### 4.1. Endothelial Regeneration Is Preserved in *Glp1r*^−/−^ Mice

GLP1 appears to have a protective influence on the vascular system. Liu et al. postulate several mechanisms for GLP1-mediated improvement in endothelial function. These include upregulation of NO synthesis, reduction of reactive oxygen species (ROS), and cyclooxygenase 2 expression (COX2) [34]. Similar effects like reduced vascular inflammation, lower ROS expression, and improved endothelial dysfunction were described previously by Ceriello et al. as well [13]. Furthermore, Torimoto et al. reported GLP1-mediated reduced postprandial triglyceride levels (TG) in human T2DM patients and therefore suggested indirect antiatherogenic effects of GLP1 [35]. Hence, it seems likely that the sitagliptin-mediated improved endothelial regeneration may potentially reflect contributions from GLP1. In contrast to these data, we demonstrated that inhibition of DPP4 still accelerates endothelial regeneration in *Glp1r*^−/−^ mice. Those findings are in line with our earlier studies in a C57Bl/6 wild-type mouse model. Besides an improved reendothelialization in sitagliptin-treated mice, we could decrypt the SDF1-CXCR4 axis as the underlying mechanism of this effect by using an additional treatment with the CXCR4 receptor blocker AMD3100 in both mouse strains [3]. Moreover, Ohnishi et al. detected an increased recruitment of endothelium-like cells via the SDF1-CXCR4 axis after acute ischemic kidney injury to peritubular vessels [36]. Additionally, deficiency of the CXCR4 receptor leads to an impaired endothelial regeneration after vascular injury [20]. This confirms that enhanced recruitment of circulating progenitor cells is the key mechanism for gliptin-induced acceleration of endothelial regeneration in this situation of acute vascular injury and shows that endothelial regeneration is preserved in the setting of murine GLP1R-deficiency—at least in the absence of further metabolic disturbances.

### 4.2. Circulating Progenitor Cells Are Responsible for the Improved Endothelial Regeneration

As we previously demonstrated, ciPC as well as monocyte/macrophages are involved in vascular regeneration [2, 3]. In a C57Bl/6 mouse model, we observed an improved endothelial regeneration from the borders of the lesions after acute arterial injury in sitagliptin-treated mice. DPP4 inhibition enhanced the recruitment of ciPC via the SDF1-CXCR4 axis to the injured sites, where the ciPC presumably induced proliferation of resident endothelial cells. This assumption is further supported by the pattern of progressive reendothelialization from the margins of the injured sites. Hence, a transdifferentiation of the recruited ciPC seems to be unlikely [3].

In this project, we demonstrated an increased recruitment of CXCR4^+^CD133^+^ and CXCR4^+^Flk-1^+^ ciPC in sitagliptin-treated *Glp1r*^−/−^ mice. Due to an improved FACS protocol, we detected a remarkably higher percentage of CXCR4^+^CD133^+^ and CXCR4^+^Flk-1^+^ ciPC. However, the relative increase of CXCR4^+^ subtypes after treatment with sitagliptin is comparable in both our studies.

Notably, GLP1 receptor signaling is abolished in this mouse model but endothelial regeneration is still accelerated after sitagliptin treatment. In addition, DPP4 inhibition also increases local SDF1 levels and enhances the SDF1-CXCR4 axis after ischemia-reperfusion injury in mice after lung transplantation [37], which confirms the ciPC recruitment via this pathway after tissue injury. Finally, by using the CXCR4 receptor blocker AMD3100, we established the importance of SDF1 mechanistically in the *Glp1r*^−/−^ mouse model. We could show that AMD3100 treatment alone (*Glp1r^−/−^* and wild-type) and in combination with sitagliptin (*Glp1r^−/−^*) inhibited endothelial healing as compared to the placebo-treated mice. These results further support our theory that inhibition of the CXCR4 receptor on the progenitor cell surface leads to a reduced recruitment of these regenerative cells into the injured tissue. Hence, the increased proportion of recruited ciPC is likely responsible for the increased regeneration capacity after acute vascular injury.

Furthermore, we and others have shown that monocytes express CXCR4 on their surface and that SDF1-CXCR4 signaling is involved in monocyte recruitment and macrophage polarization [2, 3, 38]. Sitagliptin treatment primed monocyte differentiation towards the regenerative M2 subtype instead of the inflammatory M1 subtype and led to a reduced atherosclerosis progression (chronic vascular injury) in ApoE knockout mice [2]. Chatterjee et al. substantiate our findings analyzing cultured human monocytes. They deciphered CXCL12- (SDF1) CXCR4 signaling as a pathway which first has the ability to recruit monocytes and second primes them towards the differentiation to the M2 subtype in vitro [38]. However, this mechanism appears to be more relevant in a chronic setting of high cholesterol-induced atherogenesis, where M2-type macrophages are involved in reverse cholesterol transport [39–41]. Instead, in our acute model of vascular injury, in the absence of metabolic disturbances, we did not observe differences in macrophage numbers or polarization.

### 4.3. Gliptin-Mediated Endothelial Regeneration Is Independent from the GLP1 Receptor

Our data indicate no difference in the acceleration of endothelial regeneration by sitagliptin in mice with intact as compared to deficient GLP1 receptor signaling. These findings further highlight the lack of importance of the GLP-1R in endothelial healing, consistent with our findings that the effects mediated by DPP4 inhibition are SDF1- but not GLP1R-dependent [3, 29, 42]. Eriksson et al. showed that exendin-4, a GLP1 receptor agonist, did not have a positive effect on reendothelialization after balloon injury of the carotid artery in Sprague-Dawley rats [43] which further supports our findings. Using the Glp1r^−/−^ mouse model, we could not only confirm the findings from Eriksson et al. but could also show that the missing GLP1 receptor does not have an additional negative influence on endothelial regeneration. As stimulation of the GLP1 receptor as well as the knockout of the GLP1 receptor does not lead to an alteration of reendothelialization, we can conclude that GLP1 has no relevant influence on endothelial cell proliferation after acute vascular injury in the absence of additional metabolic dysregulation.

Consistent with our previous studies in C57Bl/6 wild-type mice [3], we could not detect any differences in neointimal formation in the *Glp1r*^−/−^ mouse model 28 days after carotid injury procedure. This data is particularly important, since former studies by Zernecke et al. postulated that an enhanced SDF1-CXCR4 axis promotes neointimal hyperplasia by recruitment of bone marrow-derived smooth muscle cells (BM-SMC) [15]. Karshovska et al. further examined this controversy and identified an increased HIF1*α* expression after vascular injury as a stimulus for both the upregulated SDF1 expression in platelets and the resulting recruitment of BM-SMC [17]. A sole increase of SDF1 concentrations by inhibition of its degradation thus avoids the problem of neointimal hyperplasia as SDF1 is located downstream in the HIF1*α*-SDF1 signaling cascade. This explains why treatment with sitagliptin has no adverse effects on neointimal formation after acute endothelial injury.

### 4.4. SDF1 Has a Direct Proproliferative Effect

In addition to the gliptin effect on endothelial regeneration via the SDF1-CXCR4 axis in the *Glp1r*^−/−^ mouse model, we demonstrated a direct proregenerative effect of SDF1 on human vascular endothelial cells (HUVECs) using a scratch assay. This assay measures a combination of three processes regulating endothelial healing also in vivo: (inhibition of) apoptosis, proliferation, and migration. This suggests that, in conjunction with the enhanced recruitment of ciPC, locally increased SDF1 levels might further promote endothelial regeneration after acute vascular injury. Although data on proliferative SDF1 effects is incomplete, it is known that SDF1 has positive effects on angiogenesis and endothelial cell migration [44, 45]. A direct proproliferative SDF1 effect was confirmed, for example, for astrocytes [46], but not yet for human endothelial cells in microvasculature. Neuhaus et al. previously described an increased proliferation of human aortic endothelial cells via SDF1 induced enhanced VEGF expression [44]. This synergism of SDF1 and VEGF might be a possible underlying mechanism for the increased HUVEC proliferation and improved scratch closure promoted by high SDF1 concentrations in our experiment. Further studies are needed in the future for a detailed elucidation of the direct and indirect SDF1-mediated proproliferative effects on the diseased endothelium.

## 5. Conclusion

Pharmacological inhibition of the enzyme DPP4 reduces SDF1 cleavage and therefore increases the local SDF1 concentration after vascular injury and supports the SDF1-CXCR4-dependent recruitment of circulating progenitor cells. Using various methods, we confirmed the SDF1-CXCR4 axis as a crucial signaling pathway for endothelial regeneration after acute vascular injury, not only in wild-type animals but also in the absence of functional GLP1 receptor signaling.

Our study provides novel insight into the direct and indirect effects of SDF1-CXCR4 signaling on endothelial regeneration. Sitagliptin acted through both, the recruitment of supportive cell types and directly on endothelial cell regenerative capacity. These findings further support the assumption that endothelial healing occurs from the borders of the injured area by proliferation of resident endothelial cells [3].

In addition, we newly show that the proregenerative effects of sitagliptin are preserved in the absence of the GLP1 receptor. In this situation of acute vascular injury in the absence of dysglycemia, GLP1 has no relevant influence on endothelial cell proliferation and gliptins act on the vasculature independently from the incretin GLP1. We could identify the SDF1-CXCR4 axis as the main factor of gliptin-mediated endothelial regeneration after acute vascular injury.

Our data therefore underline the therapeutic potential of gliptin treatment after acute vascular injury that occurs in vascular diseases or vascular interventional therapies. Quick reendothelialization is essential for the prevention of vascular thromboses, neointima formation, in-stent restenoses, and vascular remodeling. Thus, as a well-tolerated class of drugs with proven cardiovascular safety, gliptins may represent potential candidates for therapy after acute vascular injury. Notably, sitagliptin has also been shown to robustly prevent SDF-1 degradation in human subjects with T2DM [47]. Large animal experiments and randomized human clinical trials should therefore examine the therapeutic potential of DPP4 inhibitors in vascular diseases, to ensure drug efficiency and to prepare the way for a use of DPP4 inhibitors for vascular regeneration.
